# Metagenomic next‐generation sequencing in the early diagnosis of leptospirosis infection presenting as acalculous cholecystitis and septic shock in a non‐epidemic area after typhoons: A case report

**DOI:** 10.1002/ccr3.9095

**Published:** 2024-06-21

**Authors:** Xueying Han, Bihan Huang, Lilei Yang, Jinglan Wu, Haigang Zhang

**Affiliations:** ^1^ Department of Critical Care Medicine Shenzhen Nanshan Hospital Shenzhen China; ^2^ Department of Critical Care Medicine The 6th Affiliated Hospital of Shenzhen University Shenzhen China; ^3^ Department of Cardiology Shenzhen Nanshan Hospital Shenzhen China; ^4^ Department of Cardiology The 6th Affiliated Hospital of Shenzhen University Shenzhen China

**Keywords:** case report, cholecystitis, leptospirosis, metagenomic next‐generation sequencing, septic shock

## Abstract

**Key Clinical Message:**

Leptospirosis is an important zoonosis worldwide. Due to nonspecific clinical manifestation and poor recognition in non‐epidemic area, there is often a delay in diagnosis and treatment. Early diagnosis from Metagenomic next‐generation sequencing test is crucial for timely intervention.

**Abstract:**

We presented a case of a 19‐year‐old male patient who developed leptospirosis infection characterized by acalculous cholecystitis and septic shock after typhoon events. Metagenomic next‐generation sequencing (mNGS) helped to early diagnose leptospirosis infection. Finally, the patient achieved full recovery following the antibiotic treatment in addition to supportive care and was discharged.

## 1 INTRODUCTION

Leptospirosis is prevalent in underdeveloped regions in tropical and subtropical areas.^1^ The transmission of the leptospirosis commonly occurs through contact with contaminated urine, water, or soil.^2^ Leptospirosis infection are presented with complex and diverse symptoms. Therefore, leptospirosis infection is commonly misdiagnosed, leading to high mortality rate.^3^ With the rapid urbanization, leptospirosis infection in Chinese cities is extremely uncommon.^4^ Consequently, leptospirosis is not typically considered as the primary diagnosis. However, there are potential risk of leptospirosis infection after typhoon events.^5^, ^6^, ^7^ In this report, we present a case of a patient who developed leptospirosis infection presented with acalculous cholecystitis and septic shock in a non‐epidemic region following typhoon events.

## 2 CASE PRESENTATION

### 2.1 Medical history and examination

A 19‐year‐old male patient presented with high fever, abdominal pain, and repeated episodes of syncope in the initial days of September 2023 after powerful typhoon Haikui and typhoon Saola. The patient, who was previously in good health, stated that he had not traveled to any regions of endemic. The patient's physical examination indicated a body temperature of 39.2°C, a pulse rate of 134 beats per minute, blood pressure of 80/47 mmHg, respiratory rate of 22 breaths per minute, and oxygen saturation of 97% while breathing room air. The cardiac and pulmonary assessments demonstrated normal heart sounds without any murmurs, as well as clear lung sounds. The examination of the abdomen revealed significant tenderness in the right upper quadrant, accompanied by a positive Murphy's sign.

### 2.2 Method (differential diagnosis, investigations, and treatment)

**FIGURE 1:**
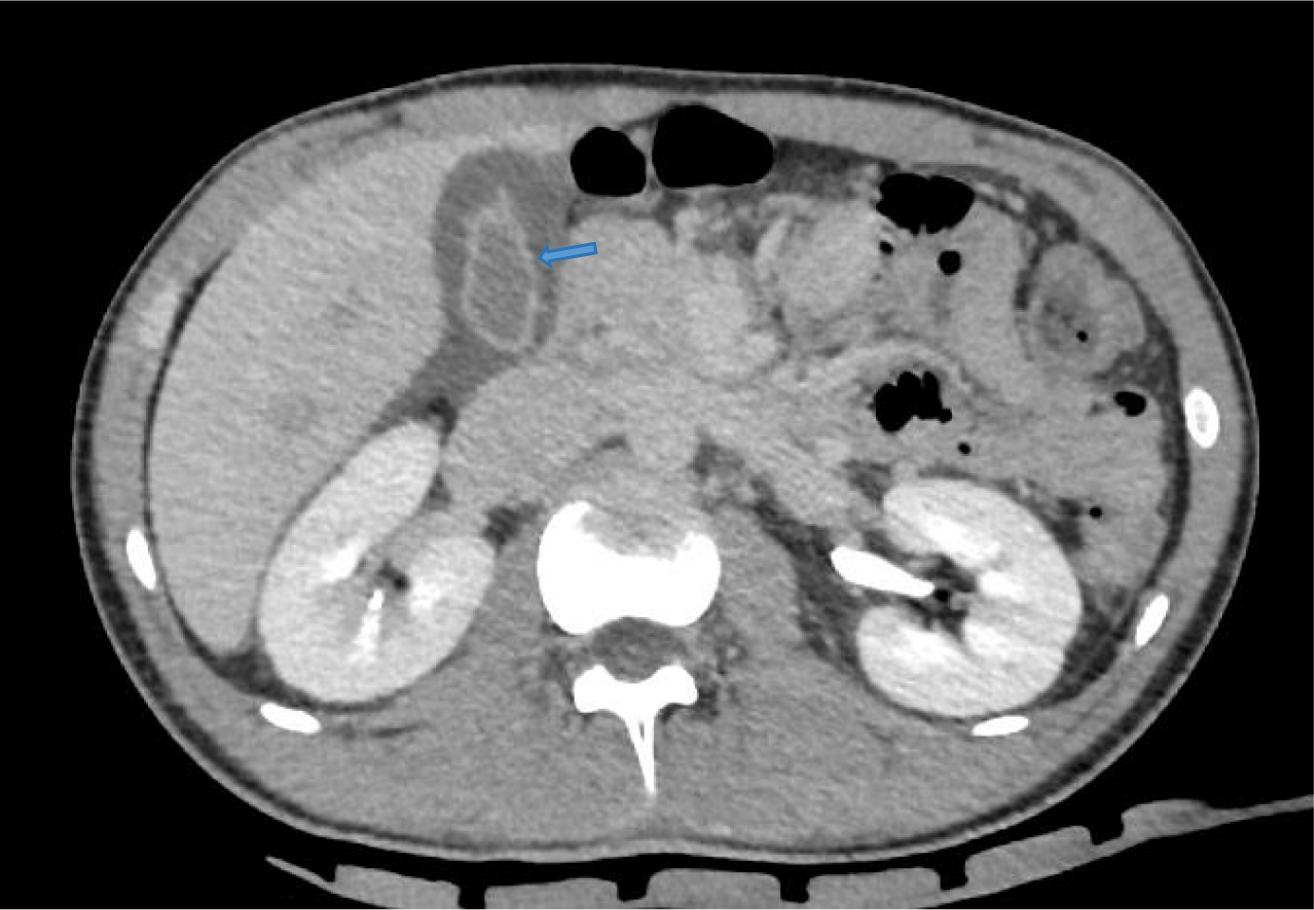
The CT scan of the abdomen revealed a thickened and edematous gallbladder wall, with no presence of gallstones, which were indicative of acalculous cholecystitis.

The laboratory findings revealed a white blood cell count of 6.8 × 10^9^/L, platelet count of 138 × 10^9^/L, neutrophil percentage of 94.8%, hemoglobin level of 112 g/L, C‐reactive protein concentration of 124.5 mg/L, procalcitonin level of 2.791 ng/mL, and interleukin‐6 concentration exceeding 5000 pg/mL. The biochemical analysis results indicated a creatinine level of 78.9umol/L, total bilirubin level of 8.8 μmol/L, alanine aminotransferase level of 17 U/L, and aspartate aminotransferase level of 32 U/L. The chest computed tomography (CT) indicated no abnormalities was detected. The abdominal CT indicated the presence of acalculous cholecystitis (Figure 1). The blood cultures conducted to detect microorganisms, as well as the tests performed to identify viral hepatitis, HIV, syphilis, clonorchis sinensis, influenza viruses, and COVID‐19 were negative. Further investigations were conducted to explore the underlying cause of the patient's infection. Hence, peripheral blood metagenomic next‐generation sequencing (mNGS) testing was conducted. The mNGS detected the presence of Leptospira interrogans (sequence number 4174). After conducting a thorough patient history, the leptospirosis infection was considered due to the patient's report of significant rainfall and extensive water accumulation around his residence following typhoons. And he engaged in recreational activities on the beach after the typhoons.

### 2.3 Conclusion and results (outcome and follow‐up)

Based on the thorough epidemiological exposure history and the results obtained from mNGS, a treatment regimen consisting of intravenous administration of ceftriaxone at a dosage of 2 g every 12 h, and doxycycline at a dosage of 0.1 g every 12 h, was administrated. The patient's condition gradually improved, C‐reactive protein, procalcitonin level, and interleukin‐6 concentration returned to normal. Antibiotic and supportive care was continued for a duration of 7 days. Finally, the patient achieved full recovery and subsequently discharged.

This case report underscores leptospirosis infection is commonly misdiagnosed in non‐epidemic area due to nonspecific clinical manifestation and poor recognition. It is difficult to diagnose by conventional methods. This case report underscores the early diagnostic value of mNGS is crucial for timely specific antibiotic treatment and supportive care.

## 3 DISCUSSION

Leptospirosis is a zoonotic disease. Leptospirosis infection has been overlooked or given insufficient attention in the cities of China.^8^ However, leptospirosis infection are commonly reported following typhoons or severe flooding in many regions.^5^, ^6^, ^9^ The transmission of the leptospirosis commonly occurs through contact with contaminated urine, water, or soil.^2^ In this case, significant rainfall and extensive water accumulation around the residence of the patient following typhoons and he engaged in recreational activities on the beach after the typhoons, which likely caused infection of Leptospira.

Leptospirosis typically presented with fever, muscle pain, and headache in a majority of patients. Conjunctival suffusion, characterized by prominent redness of the conjunctiva, is observed in approximately 50% of cases and is considered a distinctive feature of the leptospirosis infection.^5^, ^9^ Weil's syndrome, characterized by the presence of three key symptoms: conjunctival injection, jaundice, and acute kidney injury, is a well‐known clinical manifestation in the severe cases of leptospirosis infection.^10^ This patient initially presented with a high fever and abdominal pain. The abdominal CT revealed the presence of cholecystitis. These symptoms can be easily overlooked by clinicians and often poses challenges in early diagnosis.

To the best of our knowledge, there are limited literatures regarding leptospirosis infection presented with cholecystitis as the initial manifestation.^11^, ^12^ A proposed mechanism is an immune‐mediated response to leptospira, which subsequently leads to endothelial damage and submucosal edema of the gallbladder.^12^

There are various diagnostic methods for the detection of leptospirosis. The Leptospira culture is a diagnostic test and is widely regarded as the definitive method for identifying the causative agent of leptospirosis. However, the detection of Leptospira through Leptospira culture can be time‐consuming, usually about 13 weeks, and require specific types of growth media. The role of culture in clinical diagnosis is limited.^13^, ^14^ An enzyme‐linked immunosorbent assay has been developed to detect specific antibodies in leptospirosis, usually after 1 week, including IgM and IgG. However, this method do not facilitate the early diagnosis of the infection and has a tendency to false‐positive results.^15^ In addition to traditional techniques, patients may benefit from metagenomic next‐generation sequencing (mNGS). The mNGS technique is a fast and efficient method that enables the comprehensive analysis of nucleotide sequences in a given sample. In recent years, mNGS has emerged as a valuable tool in the microbiology examinations.^16^, ^17^ The mNGS holds great potential as a highly sensitive approach for the identification of specific microorganisms and the early detection of uncertain, infrequent, or newly emerging microorganisms. The mNGS can accurately and effectively identify leptospira DNA during the infection. In this case, the test of mNGS on the patient's blood sample successfully identified the leptospires infection. This timely and accurate detection played a crucial role in preventing misdiagnosis.

## 4 CONCLUSION

Medical professionals should be aware of the potential Leptospirosis infection in patients presented with acalculous cholecystitis and septic shock after typhoon events. Furthermore, mNGS is a valuable diagnostic tool in the timely identification of leptospirosis infections, particularly in patients who presented with the non‐specific symptoms.

## AUTHOR CONTRIBUTIONS

**Xueying Han:** Writing – review and editing. **Bihan Huang:** Writing – original draft. **Lilei Yang:** Writing – review and editing. **Jinglan Wu:** Writing – review and editing. **Haigang Zhang:** Writing – original draft.

## FUNDING INFORMATION

This study was supported by the Health Science and Technology Program of Nanshan District (grant number NS2024023).

## CONFLICT OF INTEREST STATEMENT

The authors declare no competing interests.

## ETHICS STATEMENT

Not applicable.

## CONSENT

Written informed consent was obtained from the patient to publish this report in accordance with the journal's patient consent policy.

## Data Availability

All data generated or analyzed in this study are included in this published article. Additional inquiries can be addressed to the corresponding author.
